# CanIsoNet: a database to study the functional impact of isoform switching events in diseases

**DOI:** 10.1093/bioadv/vbad050

**Published:** 2023-04-17

**Authors:** Tülay Karakulak, Damian Szklarczyk, Cemil Can Saylan, Holger Moch, Christian von Mering, Abdullah Kahraman

**Affiliations:** Institute of Molecular Life Sciences, University of Zurich, Winterthurerstrasse 190, Zurich, 8057, Switzerland; Department of Pathology and Molecular Pathology, University Hospital Zurich, Schmelzbergstrasse 12, Zurich, 8091, Switzerland; Swiss Institute of Bioinformatics, Amphipôle, Quartier UNIL-Sorge, Lausanne, 1015, Switzerland; Institute of Molecular Life Sciences, University of Zurich, Winterthurerstrasse 190, Zurich, 8057, Switzerland; Swiss Institute of Bioinformatics, Amphipôle, Quartier UNIL-Sorge, Lausanne, 1015, Switzerland; Computational Science and Engineering Department, Informatics Institute, Istanbul Technical University, Reşitpaşa, İTÜ Ayazağa Kampüsü, Istanbul, 34467, Türkiye; Department of Pathology and Molecular Pathology, University Hospital Zurich, Schmelzbergstrasse 12, Zurich, 8091, Switzerland; Faculty of Medicine, University of Zurich, Zurich, 8006, Switzerland; Institute of Molecular Life Sciences, University of Zurich, Winterthurerstrasse 190, Zurich, 8057, Switzerland; Swiss Institute of Bioinformatics, Amphipôle, Quartier UNIL-Sorge, Lausanne, 1015, Switzerland; Department of Pathology and Molecular Pathology, University Hospital Zurich, Schmelzbergstrasse 12, Zurich, 8091, Switzerland; Swiss Institute of Bioinformatics, Amphipôle, Quartier UNIL-Sorge, Lausanne, 1015, Switzerland; School for Life Sciences, Institute for Chemistry and Bioanalytics, University of Applied Sciences Northwestern Switzerland, Hofackerstrasse 30, Muttenz, 4132, Switzerland

## Abstract

**Motivation:**

Alternative splicing, as an essential regulatory mechanism in normal mammalian cells, is frequently disturbed in cancer and other diseases. Switches in the expression of most dominant alternative isoforms can alter protein interaction networks of associated genes giving rise to disease and disease progression. Here, we present CanIsoNet, a database to view, browse and search isoform switching events in diseases. CanIsoNet is the first webserver that incorporates isoform expression data with STRING interaction networks and ClinVar annotations to predict the pathogenic impact of isoform switching events in various diseases.

**Results:**

Data in CanIsoNet can be browsed by disease or searched by genes or isoforms in annotation-rich data tables. Various annotations for 11 811 isoforms and 14 357 unique isoform switching events across 31 different disease types are available. The network density score for each disease-specific isoform, PFAM domain IDs of disrupted interactions, domain structure visualization of transcripts and expression data of switched isoforms for each sample is given. Additionally, the genes annotated in ClinVar are highlighted in interactive interaction networks.

**Availability and implementation:**

CanIsoNet is freely available at https://www.caniso.net. The source codes can be found under a Creative Common License at https://github.com/kahramanlab/CanIsoNet_Web.

**Supplementary information:**

Supplementary data are available at *Bioinformatics Advances* online.

## 1 Introduction

Alternative splicing is an essential mechanism to regulate the generation of various mature mRNA transcripts from a single gene. Dysregulation of the splicing mechanism can lead to expression of alternative isoforms (Scotti and Swanson, 2016). Depending on the length and composition of alternative isoforms, switching events can lead to the loss or gain of interaction domains in affected gene products leading to disruptions of cellular pathways (Marti-Solano *et al.*, 2020). The IsoformSwitchAnalyzeR software has been developed to probe these functional losses or gains of protein domains in RNAseq data (Vitting-Seerup and Sandelin, 2019), while the DIGGER database reports isoform-specific interactions on an isoform and exon level (Louadi *et al.*, 2021). However, a database or webserver that provides disease-specific isoform switching data for diverse disease types is currently missing. Here, we describe *CanIsoNet*, a database that merges disease-specific isoform data with STRING (Szklarczyk *et al.*, 2015) protein interaction networks, and pathogenic information from ClinVar (Landrum *et al.*, 2014) to visualize and identify functional impact of isoform switching events in various diseases.

## 2 Implementation

We constructed disease-specific isoform interaction networks for human and mouse by integrating RNAseq transcriptome data of different diseases with Pfam domain-domain interaction data from the 3did (v 2020_05) and the STRING (v10.0) protein–protein interaction databases based on methods described earlier (Kahraman *et al.*, 2020) (see Supplementary Information). For the first version of CanIsoNet, FASTQ files from RNAseq experiments on Alzheimer's Disease (GEO: GSE203206), Parkinson's Disease (GEO: GSE128177), Behçet's disease (GEO: GSE205867) and Opioid-induced Hyperalgesia (ENA: PRJNA433105) disease including normal samples, were downloaded from the Gene Expression Omnibus (https://www.ncbi.nlm.nih.gov/geo) or ENA databases (https://www.ebi.ac.uk/ena). The transcript abundances were estimated using Kallisto (Bray *et al.*, 2016) on transcripts from the ENSEMBL database (Ensembl Mouse genome: GRCm38.p6, Ensembl Human genome: GRCh37.p13).Transcript Per Million (TPM) values were collected for each disease and normal cases. Based on the TPM values disease-specific Most Dominant Transcripts (dMDT) were called and their impact on our isoform interaction networks was assessed (see Supplementary Information) (Kahraman *et al.*, 2020).

The webservice to CanIsoNet was implemented using Python Flask (Python version 3.7) with JavaScript/AJAX extensions, Bootstrap version 4.3.1 for the front-end and a MySQL database at the backend. The Python Plotly library was used for plotting. All plots are zoomable and downloadable. The STRING network was constructed using the STRING API and the SVG graphics were adjusted using JavaScript to visualize interaction disruptions. Using the same JavaScript approach, we highlighted disease related genes from ClinVar (data downloaded July 13, 2022) in the network visualization. Domain structures of transcripts were generated by the wiggleplotr R package (Alasoo *et al.*, 2018). Anatograms were produced by the gganatogram Rshiny app (Maag, 2018) or drawn manually, if the tissue was not available in gganatogram. CanIsoNet can be browsed by disease names or searched by gene or isoform identifiers. Files including isoform interaction networks, STRING interaction density scores or interaction disruption in dominant transcripts for both human and mouse isoforms are available for download from https://www.caniso.net/download.

## 3 Results

CanIsoNet currently stores splicing information for a total of 7528 genes, 11 811 isoforms and 14 357 unique isoform switching events across 27 cancer types, Alzheimer's Disease, Parkinson's disease, Behçet's Disease and Opioid-induced Hyperalgesia. All datasets can be browsed and downloaded via dedicated drop-down menus or the download page.

dMDT information in CanIsoNet website are presented at five nested levels. The first layer is the homepage, which lists all disease types. From the home page the user can connect to the second layer corresponding to disease-specific pages giving statistics on dMDT of a sample. The disease-specific page links to the third level of gene-specific sites that provide information on dMDT of a gene. From the gene-specific sites the user can enter the fourth level of the database in which transcript specific-pages show annotations on interaction losses for each dMDT. The fifth level are sample-specific transcript pages that provide expression values for a dMDT in a specific sample.

A table listing dMDT unique to single diseases can be downloaded via the main menu of CanIsoNet. These disease-type-specific MDT can serve as putative candidates for potential diagnostic or therapeutic biomarkers (Dong and Chen, 2020; Zhao *et al.*, 2013). For example, the 489 AA long isoform of the Calcium-binding mitochondrial carrier protein SLC25A25-005, was found in 11 of 39 Alzheimer’s disease samples but not in any other disease in CanIsoNet. Its dysfunction might play role in the pathogenesis of Alzheimer’s disease (Babenko *et al.*, 2018). It’s important to note however that the uniqueness relates to the current database version of CanIsoNet. The table content is likely going to change in future versions of CanIsoNet when additional diseases are added to the database.

Overall, CanIsoNet is the first isoform-specific interaction network webserver for disease-specific isoforms. With its user-friendly interface and its rich annotations, CanIsoNet supports the discovery of functional and pathogenic isoform switching events. Upon request, new datasets can be analysed and uploaded to the CanIsoNet database. See the Contribute link on the main menu of the website, for more details. Future releases will include an automated pipeline to upload, and automatically analyse and visualize custom RNAseq data.

## 4 Use case

We will demonstrate the usage of the five nested information levels in CanIsoNet via the example of the transcript HSPH1-002, which we found as a dMDT in over 20% of Alzheimer’s disease samples. Alzheimer’s disease is a progressive brain disorder caused by neuronal damage in the brain leading to dementia, memory loss, confusion, cognitive impairment. The known diagnostic biomarkers of Alzheimer's disease are accumulated phosphorylated tau and beta-amyloid proteins (2022 Alzheimer’s Disease Facts and Figures, 2022). However, the molecular mechanisms and aetiology of Alzheimer’s disease remains unknown (Cummings *et al.*, 2019). To support the search for new biomarker and therapeutic candidates among alternative spliced isoforms, we have downloaded RNAseq data from a recent study (NCBI GEO: GSE203206) on Alzheimer’s disease and computed dMDT. All results have been uploaded to CanIsoNet and are available for further investigation by the research community (see Supplementary Information).

**Fig. 1.**
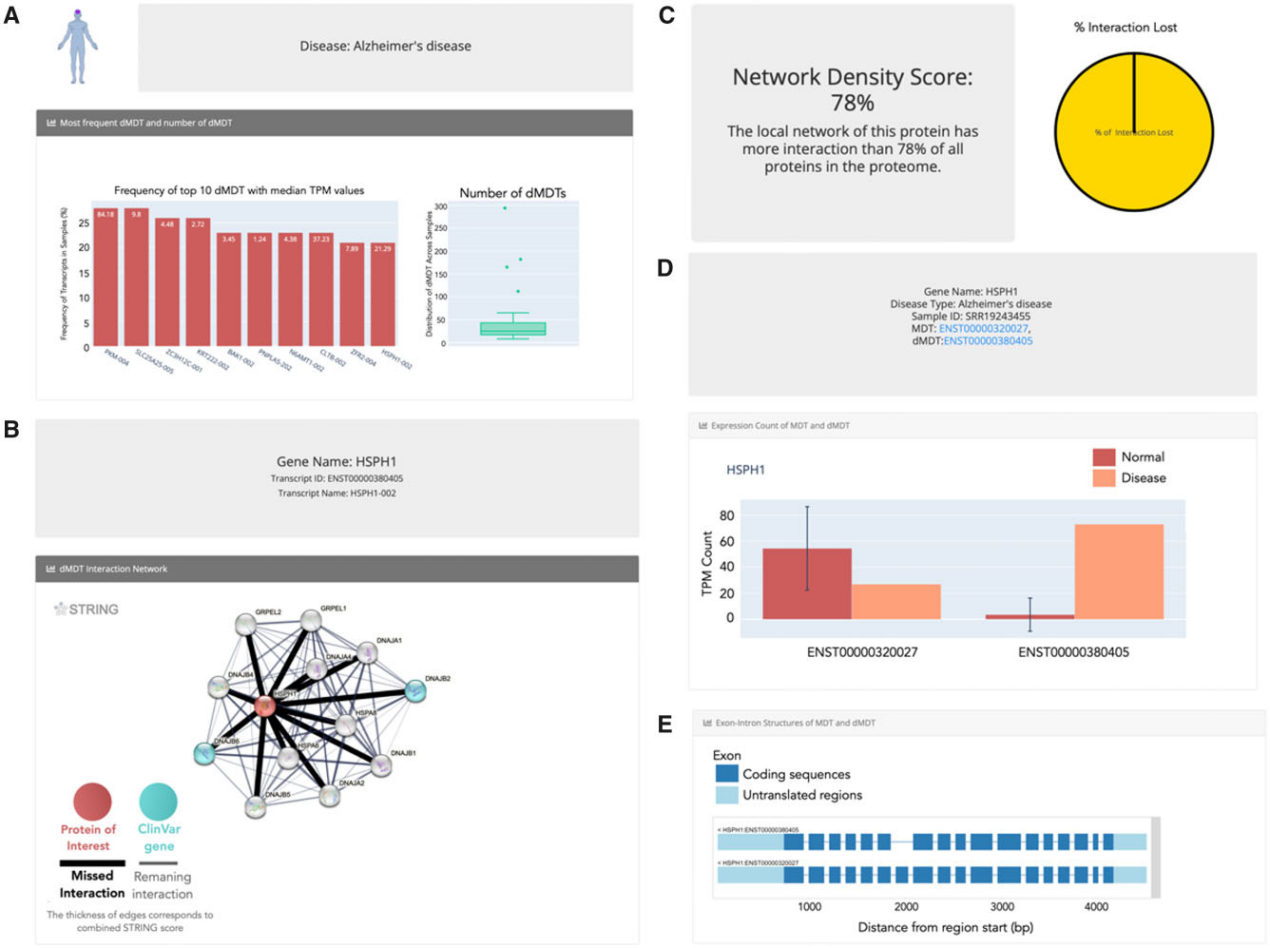
Exemplary case: (**A**) Disease page; frequency of top 10 dMDT, which are most frequently observed in Alzheimer's Disease. (**B**) Isoform page; Interaction network of the isoform HSPHS1-002. Red sphere shows the protein of interest, spheres in turquoise highlight ClinVar genes. Black line represents the lost interaction from a dMDT, while gray lines represent the remaining interactions. (**C**) Network density score and the percentage of interaction loss of the HSPH1-002. (**D**) Sample page; Median and sample specific expression level of MDT and dMDT, respectively. (**E**) Exon-intron structure of normal MDT and dMDT in a specific sample

To access the data, the user must select ‘Alzheimer’s disease’ from the disease-type table on the home page. This will open the disease-specific page for Alzheimer’s disease in CanIsoNet. At the top of the page is a barplot showing the top 10 most frequent dMDT in Alzheimer’s disease, a boxplot highlighting the number of dMDT in each sample and additional two tables (Fig. 1A). The first table lists all switching events in Alzheimer’s disease while the second table lists the total number of dMDT per sample that are visualized in the boxplot. Using the first table, the user can search for a specific isoform in the disease. For example, a switching event in the MDT of the *HSPH1* gene is among the top 10 most frequent dMDT in Alzheimer’s disease. 21% of the analysed Alzheimer’s samples show a switch between its canonical transcript HSPH1-001 (858 AA long) and its 814 AA short transcript HSPH1-002. *HSPH1* belongs to the heat-shock HSP70 protein family and was shown to play role in stabilizing misfolded proteins (Mattoo *et al.*, 2013, 110), and preventing amyloid formation (Yakubu and Morano, 2021).

Searching for the dMDT transcript ID ENST00000380405 (or HSPH1-002) in the first table on the disease-specific site opens a transcript specific-website dedicated to visualizing information on transcripts. The user can check the STRING interaction network and observe lost interactions due to domain-domain interaction losses. HSPH1-002 has 44 high-quality interactions (combined score > 900) in STRING. Of these 12 are likely physical due to known Pfam domain-domain interactions between the proteins which are amendable for interaction loss analysis due to MDT switches. Based on our analysis, the switching event would lead to the loss of all 12 physical interactions (Fig. 1B). Among the disrupted interactions are those to DNAJB2, DNAJB6 and other heat shock proteins. (Zarouchlioti *et al.*, 2018). DNAJB2 and DNAJB6 are highlighted in turquoise, which implicates that both drive disease development as described by the ClinVar database. Indeed, when clicking on both gene names in the table at the bottom of website the user is directed to the ClinVar website, where the information is given that DNAJB2 and DNAJB6 are associated with hereditary motor neuropathy and limb-girdle muscular dystrophy, respectively, which might support a potential role of *HSPH1* in Alzheimer’s disease.

Below the network representation is a table listing all lost interactions, the network density score of the protein and pie charts visualizing the percentage of lost interactions. The network density score is a weighted sum of all interactions around a protein normalized by the maximum score in the network. A score of 78% as in the case of HSPH1 (Fig. 1C), denotes that HSPH1 has more interactions in its local neighbourhood than 78% of all other proteins in the human STRING interaction network. Since essential and cancer related genes are known to form network hubs (Goh *et al.*, 2007), the network density score should help to prioritize dMDT and give an estimate on the functional impact of an interaction loss due to dMDT. Below the network density score information, is another table listing all samples in which the dMDT was identified. Clicking on SRR19243455 loads the sample-specific page of the Alzheimer sample SRR19243455, where the user finds a barplot showing the relative expression values of each dMDT and the median expression value of MDT in normal tissue samples (Fig. 1C). An exon-intron gene structure highlights differences in the exons and introns structures between the dMDT and the normal MDT (Fig. 1D).

Besides browsing through CanIsoNet, the user has the option to search for the *HSPH1* gene name or the transcript IDs HSPH1-002 or ENST00000380405 on the main page, which will lead the user directly to the gene-specific or transcript-specific websites (see above).

## Supplementary Material

vbad050_Supplementary_Data
