# Supplementary material for: CanIsoNet: a database to study the functional impact of isoform switching events in diseases
Source: Bioinform Adv. 2023 Apr 17;3(1):vbad050. doi: 10.1093/bioadv/vbad050 (PMC10133402; doi:10.1093/bioadv/vbad050)
Supplement: vbad050_Supplementary_Data [file vbad050_supplementary_data.pdf]

CanIsoNet: A Database to Study the Functional Impact of Isoform Switching Events in Diseases

Tülay Karakulak[1,2,3], Damian Szklarczyk[1,3], Cemil Can Saylan[4], Holger Moch[2,5], Christian von Mering[1,3], Abdullah Kahraman[1,2,3,6*]

[1] Institute of Molecular Life Sciences, University of Zurich, Zurich, Switzerland [2]Department of Pathology and Molecular Pathology, University Hospital Zurich, Zurich, Switzerland [3]Swiss Institute of Bioinformatics, Lausanne, Switzerland, [4]Computational Science and Engineering Department, Informatics Institute, Istanbul Technical University, Sariyer, Istanbul, 34467, Turkey, [5]Faculty of Medicine, University of Zurich, Zurich, Switzerland, [6]School for Life Sciences, Institute for Chemistry and Bioanalytics, University of Applied Sciences Northwestern Switzerland, Muttenz, Switzerland.

*Corresponding author

e-mail: abdullah.kahraman@fhnw.ch

Supplementary Method

The most dominant transcript switching events for Alzheimer's Disease, Parkinson Disease, Behçet's Disease and Opioid-induced Hyperalgesia (OIH) were computed with the CanIsoNet software pipeline (Kahraman *et al.*, 2020) with the following accession numbers: OIH GEO: GSE126662, Alzheimer's Disease: GSE203206, Parkinson Disease: GSE128177, Behcet's Disease: GSE205867. Reads were pseudo-aligned to the Ensembl Mouse genome (GRCm38.p6) for OIH and to Ensembl Human genome (GRCh37.p13) for the remaining diseases using Kallisto (Bray *et al.*, 2016). Transcripts Per Million (TPM) counts of identical cDNA sequences were merged and only the one with the longest protein isoform sequence were kept. TPM values < 2

were set to 0 given that 99% of olfactory receptor transcripts had a TPM value of < 2. The protein-protein interaction network for Mus Musculus was downloaded from the STRING database (v10.0) (Szklarczyk *et al.*, 2015). An isoform-specific protein-protein interaction network for Mus Musculus isoforms was created following methods described in (Kahraman *et al.*, 2020). PFAM domain interactions from 3did (v.2020_01) (Mosca *et al.*, 2014) were integrated with STRING interactions and alternatively spliced protein isoform sequences from the Ensembl database (GRCm38.p6). Most Dominant Transcript (MDT) switches were calculated between the following pairs: 5 morphine treated and 5 untreated mice for OIH case in Nucleus Accumbens, 5 morphine treated and 5 untreated mice for OIH case in Trigeminal Ganglia, 8 non-demented control and 39 Alzheimer's Disease samples for Alzheimer's Disease, 24 healthy and 12 Parkinson's Disease samples for Parkinson's Disease, and 10 healthy and 10 Behçet's Disease samples for Behçet's Disease. A MDT was identified for each gene if its expression was 2x higher than the 2nd most expressed transcript. For Alzheimer's Disease, Parkinson's Disease and Behçet's Disease, the original criteria for identifying disease-specific MDT was applied, i.e. dMDT are not observed as MDT in the paired normal samples. In OIH, we allowed 20% of untreated samples to share the same MDT with treated samples and relaxed the multiple testing q-value to 0.1 due to the low sample number. In total we could identify 980, 48, 52, and 13 isoform switching events in Alzheimer's Disease, Parkinson disease, Behçet's Disease and OIH cases, respectively.

REFERENCES

Bray,N.L. *et al.* (2016) Near-optimal probabilistic RNA-seq quantification. *Nat Biotechnol*, **34**, 525–527.

Kahraman,A. *et al.* (2020) Pathogenic impact of transcript isoform switching in 1,209 cancer

samples covering 27 cancer types using an isoform-specific interaction network. *Sci Rep*, **10**, 14453.

Mosca,R. *et al.* (2014) 3did: a catalog of domain-based interactions of known three-dimensional structure. *Nucl. Acids Res.*, **42**, D374–D379.

Szklarczyk,D. *et al.* (2015) STRING v10: protein–protein interaction networks, integrated over the tree of life. *Nucleic Acids Research*, **43**, D447–D452.
